# Integration of C/N-nutrient and multiple environmental signals into the ABA signaling cascade

**DOI:** 10.1080/15592324.2015.1048940

**Published:** 2016-01-19

**Authors:** Yu Lu, Junji Yamaguchi, Takeo Sato

**Affiliations:** Faculty of Science and Graduate School of Life Science; Hokkaido University; Kita-ku Sapporo, Japan

**Keywords:** abscisic acid (ABA), ABI1, carbon, nutrient availability, nitrogen, protein phosphorylation, SnRK

## Abstract

Due to their immobility, plants have developed sophisticated mechanisms to robustly monitor and appropriately respond to dynamic changes in nutrient availability. Carbon (C) and nitrogen (N) are especially important in regulating plant metabolism and development, thereby affecting crop productivity. In addition to their independent utilization, the ratio of C to N metabolites in the cell, referred to as the “C/N balance”, is important for the regulation of plant growth, although molecular mechanisms mediating C/N signaling remain unclear. Recently ABI1, a protein phosphatase type 2C (PP2C), was shown to be a regulator of C/N response in Arabidopsis plants. ABI1 functions as a negative regulator of abscisic acid (ABA) signal transduction. ABA is versatile phytohormone that regulates multiple aspects of plant growth and adaptation to environmental stress. This review highlights the regulation of the C/N response mediated by a non-canonical ABA signaling pathway that is independent of ABA biosynthesis, as well as recent findings on the direct crosstalk between multiple cellular signals and the ABA signaling cascade.

## ABI1 functions in C/N-nutrient signal transduction

Carbon and nitrogen nutrients co-operatively regulate multiple aspects of plant growth, including early post-germinative growth, true leaf expansion, root elongation, and senescence.^1,2,3,4^ Thus, in addition to the independent utilization of carbon and nitrogen, plants sense and adapt to changes in their relative balance, called the C/N response, by precise partitioning of C and N sources and fine-tuning of complex cellular metabolic activity.^5,6^ To clarify the molecular regulatory mechanisms underlying the C/N response in plants, we screened for C/N-insensitive Arabidopsis mutants using a medium containing a relatively high concentration of glucose and a limited nitrogen source (i.e., nitrate and ammonium), high C/low N stress condition. We had previously isolated a C/N insensitive mutant, *carbon/nitrogen insensitive 1-D* (*cni1-D*), which was shown to overexpress the *CNI1* gene that encodes the ubiquitin ligase ATL31.^3^ Overexpression of *ATL31* rescued plants from post-germinative development arrest induced by high C/low N stress conditions, whereas the *atl31* loss-of-function mutant showed a hypersensitive phenotype.^3^ Subsequent analyses identified 14-3-3 proteins as ubiquitination targets of ATL31^5^ and showed that the interaction between ATL31 and 14-3-3 was dependent on the phosphorylation of specific Ser/Thr residues in the C-terminal region of ATL31.^7^ These findings revealed that the unique regulatory mechanism of plant C/N-nutrient signaling acted via the ubiquitin-proteasome system combined with protein phosphorylation.^3,5,7,8^

More recently, we isolated a new C/N-insensitive Arabidopsis mutant, *cni2-D*, which overexpresses the *ABI1* gene.^9^ ABI1 is a type 2C protein phosphatase that negatively regulates abscisic acid (ABA) signaling^10^ and is a critical component of ABA signal transduction, with its phosphatase activity inhibiting several SnRK2 proteins.^11,12^ ABA and the ABA-receptor complex bind to ABI1, with ABA inhibiting ABI1 activity,^13^ resulting in the activation of SnRK2s kinase activity and ABA signal transduction. Overexpression of *ABI1* resulted in continued post-germinative growth and expansion of green-colored cotyledons under high C/low N stress conditions, whereas the loss of function mutant *abi1-2* was hypersensitive. Interestingly, ABA quantification analysis showed that the growth defect phenotype of wild-type Arabidopsis plants under high C/low N stress conditions was not associated with endogenous ABA content. Accordingly, the *abi1-1* mutant, in which mutated ABI1 is unable to bind to the ABA-receptor complex, resulting in the constitutive inactivation of SnRK2s, was not resistant to high C/low N stress. These results strongly suggest that the amount of ABA is not a predominant factor in C/N-nutrient signaling, unlike the sugar signaling cascade which is mediated by ABA signaling coupled with ABA biosynthesis.

It is also important to determine the downstream signaling pathway that mediates C/N-nutrient signaling under ABI1 regulation. The canonical ABA insensitive mutants, *abi4* and *abi5*, both of which are loss of function mutants with defects in the SnRK2 downstream transcriptional factors *ABI4* and *ABI5*, respectively, showed similar responses to C/N stress as wild-type plants. Moreover, we found that specific ABA signaling pathways are activated by C/N stress in wild-type Arabidopsis plants. The levels of expression of *RD29b*, *LEA3-4* and *TSPO* were increased in response to high C/low N stress in wild-type plant, which are significantly repressed in *ABI1* overexpressor. In contrast, other ABA marker genes, including *RAB18*, *AREB1* and *ABF3*, were not up-regulated in response to C/N stress, with the levels of expression being similar in wild-type and *ABI1* overexpressing plants. In addition, transcription analysis also showed that SnRK1, recently determined as another direct target of ABI1,^14^ is involved in C/N-nutrient signal transduction.^15^ These results indicate that C/N directly activates specific and uncanonical ABA signaling cascades under conditions of ABI1 regulation in a manner independent of ABA biosynthesis.

The molecular and physiological relationships between ABI1/CNI2 and ATL31/CNI1 remain unclear. We previously showed that *ATL31* overexpresing plants are not insensitive to exogenous ABA in the medium, suggesting that the function of ATL31 is also independent of canonical ABA perception and its signaling pathway. Additional studies are needed to better understand crosstalk between ABA and C/N-nutrient signaling pathways.

## Direct interaction between ABA signal and other cellular signaling pathway

The ABA receptors PYRs/PYLs/RCARs, PP2Cs and SnRK2s constitute the core signal transduction system that regulates plant growth in response to environmental stimuli, with this regulation being highly dependent on the presence or absence of ABA. In addition to ABA, several abiotic stresses were found to modulates PP2Cs and SnRK2s signaling activity, similarly to our finding that C/N-nutrient stress could activate specific ABA signaling pathways independent of ABA biosynthesis.^9^ Here we summarize several reports describing the alternative regulation of PP2Cs and SnRK2s function independent of ABA biosynthesis and perception (**Table 1**).

**Table 1.** Regulation of ABA signaling directly by multiple cellular signaling pathways.

Regulator	Target	Effect	Reference
H_2_O_2_	AtHAB1	Inhibits the interaction of HAB1 and SnRK2s	^16,30,31^
AtROP11	AtABI1	Protect ABI1 phosphatase activity	^17,18^
AtBIN2, AtBIL1, AtBIL2	AtSnRK2.2, AtSnRK2.3	Phosphorylates and activates SnRK2.2 and SnRK2.3	^24^
	AtABI5	Phosphorylates and stabilizes ABI5	^23^
ZmCK2	ZmSnRK2.6/OST1	Enhances the interaction between SnRK2.6/OST1 and ABI1 and degrades SnRK2.6/OST1	^26^
NO	AtSnRK2.6/OST1	S-nitrosylation and repression of SnRK2.6	^29^

At: Arabidopsis thaliana; Zm: Zea maize

Reactive oxygen species (ROS) are key secondary messengers that respond to a variety of stress conditions. Hydrogen peroxide is the major ROS produced by plants. Recently, hydrogen peroxide was found to inhibit the activity of HOMOLOGY TO ABI1 (HAB1), a PP2C protein involved in ABA signaling in Arabidopsis.^16^ Hydrogen peroxide promotes the dimerization of HAB1 and blocks the interactions between HAB1 and downstream SnRK2s by oxidizing the Cys-186 and Cys-247 residues of HAB1. These findings demonstrated that hydrogen peroxide regulates PP2C activities and mediates ABA signaling. Although ABA promotes the production of hydrogen peroxide, the inhibition of PP2C by hydrogen peroxide can occur in the absence of ABA and ABA receptor,^16^ suggesting that ABA signaling is directly regulated by hydrogen peroxide produced by other signaling cascades. ROPs are plant specific Rho-like small GTPase family proteins, with their activity dependent on alternative binding to GTP or GDP. Arabidopsis ROP11 negatively regulates ABA mediated stomatal closure, seed germination, post-germinative growth and ABA marker gene expression by interacting with and protecting ABI1 phosphatase activity.^17,18^

It had been proposed that SnRK2s activities are regulated by osmotic stress both of ABA-dependent and -independent manner. Arabidopsis SnRK2.6/OST1, a member of the SnRK2 group III subfamily, is activated in response to osmotic stress via its C-terminal domain 2 in an ABA independent manner.^19^ SnRK2s are activated even in ABA-deficient and ABA-insensitive mutants subjected to osmotic stress, and may be activated by an upstream kinase rather than conventional auto-activation.^20^ The ABA independent osmotic stress response and the ABA independent kinase pathway via SnRK2s and their downstream transcriptional factors have been thoroughly reviewed.^21,22^

Direct crosstalk between ABA and brassionsteroid (BR) has also been reported. BRASSINOSTEROID INSENSITIVE2 (BIN2) kinase, a negative regulator of BR signaling in Arabidopsis plants, and its homologues BIN2-LIKE1 (BIL1) and BIL2, directly interact with ABI5 transcription factor in the nucleus.^23^ ABI5 phosphorylation by BIN2 and its homologues stabilizes ABI5 protein, enhancing ABA signaling and inhibiting germination and post-germinative growth. BIN2 and its homologues also directly interact with and phosphorylate SnRK2.2 and SnRK2.3, activating downstream ABA signaling and inhibiting root elongation.^24^

SnRK2.6/OST1 is predominantly expressed in guard cells and contributes to the global regulation of ABA-associated stomatal closure by auto-phosphorylation in the absence of inhibition by PP2C protein. However, the existence of an upstream kinase could not be ignored, since SnRK2.6/OST1 has been detected in other tissues and plays potential role in processes other than ABA signaling, for example in sucrose metabolism.^25^ The CASEIN KINASE 2 (CK2) holoenzyme complex, which is comprised of two α-catalytic subunits and two β-regulatory subunits, was recently shown to phosphorylate SnRK2.6/OST1 in maize.^26^ This phosphorylation negatively regulates ABA signaling by enhancing the interaction between SnRK2.6/OST1 and PP2C and also promotes SnRK2.6/OST1 degradation via the 26S proteasome system.^26^ These molecular findings demonstrate that additional upstream kinases could regulate SnRK2 activity as well as ABA signaling. Nitric oxide (NO) is an important secondary messenger that regulates many aspects of plant responses, including ABA-induced stomatal closure.^27,28^ The NO dependent S-nitrosylation of SnRK2.6/OST1 at the Cys-137 residue was recently shown to negatively regulate SnRK2.6/OST1 activity and ABA mediated stomatal closure in Arabidopsis.^29^ NO accumulates in mutants lacking the *S-nitrosoglutathione reductase* (*GSNOR*) gene, resulting in impaired ABA inducible stomatal closure. Interestingly, a cysteine residue corresponding to Cys137 of SnRK2.6 is present in several yeast and human protein kinases and could be S-nitrosylated, suggesting that S-nitrosylation may be an evolutionarily conserved mechanism for regulating protein kinases.^29^

In addition to our recent finding, that C/N signals are mediated via a non-canonical ABA signaling pathway, several studies have shown crosstalk between ABA signal transduction and multiple cellular signals, suggesting that multiple environmental signals are integrated into the ABA signaling cascade. Further studies will reveal the detailed molecular basis and physiological significance of this integration mechanism, optimizing plant growth in nature.
